# Supplementary material for: Identification of predictive factors of the degree of adherence to the Mediterranean diet through machine-learning techniques
Source: PeerJ Comput Sci. 2020 Jul 27;6:e287. doi: 10.7717/peerj-cs.287 (PMC7924593; doi:10.7717/peerj-cs.287)
Supplement: Supplemental Information 2 [file peerj-cs-06-287-s002.pdf]

Patient:________________________
History number:_________________
Name:____________________
Surname:_________________
Interviewer:______________________

SUBJECTIVE OVERALL ASSESSMENT

A. BACKGROUND:

1. Weight change

- Overall loss in the past 6 months _____kg, % loss ________

- Changes in the last 2 weeks: _______increase

_______ unchanged

_______ decrease

Change in dietary intake (from normal/usual)

- No change _________

- Change Duration ______weeks

Type: ______suboptimal ________solid diet liquid diet

______ hypocaloric fluids _________fasting

Gastrointestinal symptoms (lasting > 2 weeks)

none ____anorexia ____nausea ___vomiting _____ diarrhea

4. Functional capacity

- No alteration ________

- Alteration Duration ______weeks

Intensity: Suboptimal ______work

______ No work, no bedding

______ Bed

5. Basic disease and its relationship to nutritional needs

Primary diagnosis ____

Metabolic demands (degree of stress) None____ Low_____

Intermediate___ High _____

B. PHYSICAL EXAMINATION

For each item: 0: Normal; 1+: mild; 2+: moderate; 3+: severe

- Subcutaneous fat loss (triceps, chest) ___________

- Loss of muscle mass (quadriceps, deltoids, temporal) __________

- Presence of edema (distal, sacral) _________

- Presence of ascites ________

C. VGS CLASSIFICATION (select one)

A. Well nourished (minimal or no intake restriction, minimal changes in function, stable or increased weight.)

B. Moderately malnourished (reduced intake, some changes)
and absent or scarce body mass change)

C. Severely malnourished (evident declines in intake, function, and body mass)

DATA COLLECTION NOTEBOOK.

ADULT POPULATION 40 YEARS OLD AND OVER IN THE MUNICIPALITY OF CAMBRE

Patient:_______________________
History number:________________
Name:________________________
Surname:______________________
Interviewer:____________________

Patient identification variables:

Date of birth (dd/mm/yyyyy):

Sex:

- ☐ Male
- ☐ Woman

Place:

Telephone

Level of studies:
- ☐ no studies
- ☐ Incomplete 1st degree
- ☐ 1st grade (Elementary Baccalaureate, EGB)
- ☐ 2nd grade (Bachillerato Superior, BUP, FP)
- ☐ University

Anthropometric variables:

Weight: BMI:

Size:

Waist circumference:

Hip perimeter:

Skin folds:

Tricipital: Subscapular:
Bicipital: Suprailiac:

Arm perimeter:
Calf perimeter:

Bioimpedance:

Adipose mass: Bone mass (kg):
Liquid mass: MTB of activity in Kcal:
Muscle mass:
MTB basal caloric in Kcal:

DATA COLLECTION NOTEBOOK.

ADULT POPULATION 40 YEARS OLD AND OVER IN THE MUNICIPALITY OF CAMBRE

Patient:________________________
History number:_________________
Name:_________________________
Surname:_______________________
Interviewer:______________________

Variables of cardiovascular risk:

Smoker:

- ☐ no
- ☐ ex-smoker (patient stopped smoking more than 12 months before entering the study)
- ☐ if (patient smokes or has smoked in the last 12 months, at least 1 cigarette or 1 pipe or 1 cigar per day). Fagerström test.

TAS/TAD:

	TAD	TAS
Measurement 1		
Measurement 2		

Analytical:

Previous: date _________________ ☐ no

Current: date _________________ ☐ no (***ask for it)***

EKG:

Hypertrophy of the left ventricle (Sokolow criterion):

☐ if ☐ no

Other findings:

- ☐ FA
- ☐ AV Lock:
- ☐ Extrasystolias
- ☐ Ischemia
- ☐ Necrosis

DATA COLLECTION NOTEBOOK.

ADULT POPULATION 40 YEARS OLD AND OVER IN THE MUNICIPALITY OF CAMBRE

Patient:________________________
History number:_________________
Name:_________________________
Surname:_______________________
Interviewer:_____________________

Diseases:

ILLNESS	HISTORY	TEST CHARLSON
Myocardial infarction		
Heart failure		
Peripheral vascular disease		
Cerebral vascular disease		
Dementia		
Hemiplegia		
Chronic lung disease		
Connective tissue disease		
Peptic ulcer		
Mild liver disease		
Moderate-severe liver disease (ascites and cirrhosis)		
Diabetes mellitus		
Diabetes with organic affectation		
Moderate or severe kidney failure		
Cancer, leukemia, lymphoma		
Cancer with metastasis		
AIDS		
HTA		
Alteration of lipids		
Other		

Medication:

**Category _ medication**	**Active ingredient/drug**	**Dose**	**Guideline**
☐ Oral antidiabetics			
☐ Insulin			
☐ Diuretics			
☐ IEC			
☐ ARAS			
☐ Beta-blockers			
☐ Anti-aggregants			
☐ Hypolipemics			
☐ Benzodiazepines			
☐ Neuroleptics			
☐ NSAIDS			
☐ Paracetamol			
☐ Inhalers			
☐ Osteoporosis			
☐ OTHER			

Date of interview (dd/mm/yyyyy):

Apellidos: Nombre:

Sexo: Edad: Peso, kg: Altura, cm: Fecha:

Responda a la primera parte del cuestionario indicando la puntuación adecuada para cada pregunta. Sume los puntos correspondientes al cribaje y si la suma es igual o inferior a 11, complete el cuestionario para obtener una apreciación precisa del estado nutritional.

Cribaje

A Ha perdido el apetito? Ha comido menos por faltade apetito, problemas digestivos, dificultades de masticacióno deglución en los últimos 3 meses?
0 = ha comido mucho menos
1 = ha comido menos
2 = ha comido igual ☐

B Pérdida reciente de peso (<3 meses)
0 = pérdida de peso > 3 kg
1 = no lo sabe
2 = pérdida de peso entre 1 y 3 kg
3 = no ha habido pérdida de peso ☐

C Movilidad
0 = de la cama al sillón
1 = autonomía en el interior
2 = sale del domicilio ☐

D Ha tenido una enfermedad aguda o situación de estrés psicológico en los últimos 3 meses?
0 = sí 2 = no ☐

E Problemas neuropsicológicos
0 = demencia o depresión grave
1 = demencia moderada
2 = sin problemas psicológicos ☐

F Índice de masa corporal (IMC = peso / (talla)² en kg/m²
0 = IMC <19
1 = 19 ≤ IMC < 21
2 = 21 ≤ IMC < 23.
3 = IMC ≥ 23. ☐

Evaluación del cribaje
(subtotal máx. 14 puntos) ☐☐

12-14 puntos: estado nutricional normal
8-11 puntos: riesgo de malnutrición
0-7 puntos: malnutrición

Para una evaluación más detallada, continúe con las preguntas G-R

Evaluación

G El paciente vive independiente en su domicilio?
1 = sí 0 = no ☐

H Toma más de 3 medicamentos al día?
0 = sí 1 = no ☐

I Úlceras o lesiones cutáneas?
0 = sí 1 = no ☐

J Cuántas comidas completas toma al día?
0 = 1 comida
1 = 2 comidas
2 = 3 comidas ☐

K Consume el paciente
- productos lácteos al menos una vez al día? sí ☐ no ☐
- huevos o legumbres 1 o 2 veces a la semana? sí ☐ no ☐
- carne, pescado o aves, diariamente? sí ☐ no ☐

0.0 = 0 o 1 síes
0.5 = 2 síes
1.0 = 3 síes ☐.☐

L Consume frutas o verduras al menos 2 veces al día?
0 = no 1 = sí ☐

M Cuántos vasos de agua u otros líquidos toma al día? (agua, zumo, café, té, leche, vino, cerveza...)
0.0 = menos de 3 vasos
0.5 = de 3 a 5 vasos
1.0 = más de 5 vasos ☐.☐

N Forma de alimentarse
0 = necesita ayuda
1 = se alimenta solo con dificultad
2 = se alimenta solo sin dificultad ☐

O Se considera el paciente que está bien nutrido?
0 = malnutrición grave
1 = no lo sabe o malnutrición moderada
2 = sin problemas de nutrición ☐

P En comparación con las personas de su edad, cómo encuentra el paciente su estado de salud?
0.0 = peor
0.5 = no lo sabe
1.0 = igual
2.0 = mejor ☐.☐

Q Circunferencia braquial (CB en cm)
0.0 = CB < 21
0.5 = 21 ≤ CB ≤ 22
1.0 = CB > 22 ☐.☐

R Circunferencia de la pantorrilla (CP en cm)
0 = CP < 31
1 = CP ≥ 31 ☐

Evaluación (máx. 16 puntos) ☐☐.☐
Cribaje ☐☐.☐
Evaluación global (máx. 30 puntos) ☐☐.☐

Evaluación del estado nutricional

De 24 a 30 puntos ☐ estado nutricional normal
De 17 a 23.5 puntos ☐ riesgo de malnutrición
Menos de 17 puntos ☐ malnutrición

Ref Vellas B, Villars H, Abellan G, et al. *Overview of the MNA® - Its History and Challenges.* J Nut Health Aging 2006 ; 10 : 456-465.
Rubenstein LZ, Harker JO, Salva A, Guigoz Y, Vellas B. *Screening for Undernutrition in Geriatric Practice : Developing the Short-Form Mini Nutritional Assessment (MNA-SF).* J. Geront 2001 ; 56A : M366-377.
Guigoz Y. *The Mini-Nutritional Assessment (MNA®) Review of the Literature - What does it tell us?* J Nutr Health Aging 2006 ; 10 : 466-487.
® Société des Produits Nestlé, S.A., Vevey, Switzerland, Trademark Owners
© Nestlé, 1994, Revision 2006. N67200 12/99 10M
Para más información: www.mna-elderly.com

**Eating Disorder Inventory Subscale (EDI-IC): bodily dissatisfaction**	Patient:________________________<br>History number:________________<br>Name:_________________________<br>Surname:______________________<br>Interviewer:_____________________

Instructions

Below you will find some sentences that are commonly used to measure the patient's dissatisfaction with the general shape of their body or with that of those parts of their body that most concern those who suffer from eating disorders (stomach, hips, thighs, buttocks ...). Mark with an X what best indicates how you feel right now.

	Always	Almost always	Often	Sometimes	Almost never	Never
1. I think my stomach is too big						
2. I think my thighs are too thick.						
3. I think my stomach is the right size.						
4. I'm satisfied with my figure.						
5. I like the shape of my ass.						
6. I think my hips are too wide.						
7. I think the size of my thighs is adequate.						
8. I think my ass is too big.						
9. I think my hips are the right size.						

Eating Disorder Inventory Subscale (EDI-O): obsession with thinnessw

Instructions

The elements of this scale refer to concern for weight, diets and fear of gaining weight. Mark with an X what best indicates how you feel right now.

	Always	Almost always	Often	Someti mes	Almos t never	Never
I eat sweets and carbohydrates without feeling nervous.						
2. I'm thinking of going on a diet						
3. I feel very guilty when I eat too much						
4. I'm terrified of the idea of getting fat.						
5. I exaggerate or overemphasize weight						
6. I'm obsessed with the desire to be thinner.						
7. If I gain a kilo of weight, I'm afraid I'll continue to gain weight.						

Subjective perception of weight:

I consider my weight to be:

A) higher than normal
B) normal
C) lower than normal

DATA COLLECTION NOTEBOOK.

ADULT POPULATION 65 YEARS OLD AND OVER IN THE MUNICIPALITY OF CAMBRE

Patient:________________________
History number:_________________
Name:_________________________
Surname:_______________________
Interviewer:_____________________

Patient identification variables:

Date of birth (dd/mm/yyyyy):

Sex:

- ☐ Male
- ☐ Woman

Place:

Telephone

Level of studies:
- ☐ no studies
- ☐ Incomplete 1st degree
- ☐ 1st grade (Elementary Baccalaureate, EGB)
- ☐ 2nd grade (Bachillerato Superior, BUP, FP)
- ☐ University

Anthropometric variables:

Weight: BMI:

Size:

Waist circumference:

Hip perimeter:

Skin folds:

Tricipital: Subscapular:
Bicipital: Suprailiac:

Arm perimeter:
Calf perimeter:

Bioimpedance:

Adipose mass: Bone mass (kg):
Liquid mass: MTB of activity in Kcal:
Muscle mass:
MTB basal caloric in Kcal:

DATA COLLECTION NOTEBOOK.

ADULT POPULATION 65 YEARS OLD AND OVER IN THE MUNICIPALITY OF CAMBRE

Patient:________________________
History number:________________
Name:_________________________
Surname:_______________________
Interviewer:_____________________

Variables of cardiovascular risk:

Smoker:

☐ no

☐ ex-smoker (patient stopped smoking more than 12 months before entering the study)

☐ if (patient smokes or has smoked in the last 12 months, at least 1 cigarette or 1 pipe or 1 cigar per day). Fagerström test.

TAS/TAD:

	TAD	TAS
Measurement 1		
Measurement 2		

Analytical:

Previous: date _______________ ☐ no

Current: date _______________ ☐ no (***ask for it)***

Physical activity (more than 30 minutes of moderate-intense physical activity more than 5 days a week):

☐ if ☐ no

EKG:

Hypertrophy of the left ventricle (Sokolow criterion):

☐ if ☐ no

Other findings:

☐ FA
☐ AV Lock:
☐ Extrasystolias
☐ Ischemia
☐ Necrosis

DATA COLLECTION NOTEBOOK.

ADULT POPULATION 65 YEARS OLD AND OVER IN THE MUNICIPALITY OF CAMBRE

Patient:________________________
History number:_________________
Name:_________________________
Surname:_______________________
Interviewer:______________________

Diseases:

ILLNESS	HISTORY	TEST CHARLSON
Myocardial infarction		
Heart failure		
Peripheral vascular disease		
Cerebral vascular disease		
Dementia		
Hemiplegia		
Chronic lung disease		
Connective tissue disease		
Peptic ulcer		
Mild liver disease		
Moderate-severe liver disease (ascites and cirrhosis)		
Diabetes mellitus		
Diabetes with organic affectation		
Moderate or severe kidney failure		
Cancer, leukemia, lymphoma		
Cancer with metastasis		
AIDS		
HTA		
Alteration of lipids		
Other		

Medication:

**Category _ medication**	**Active ingredient/drug**	**Dose**	**Guideline**
☐ Oral antidiabetics			
☐ Insulin			
☐ Diuretics			
☐ IEC			
☐ ARAS			
☐ Beta-blockers			
☐ Anti-aggregants			
☐ Hypolipemics			
☐ Benzodiazepines			
☐ Neuroleptics			
☐ NSAIDS			
☐ Paracetamol			
☐ Inhalers			
☐ Osteoporosis			
☐ OTHER			

Date of interview (dd/mm/yyyyy):
